# Angiotensin II directly induces muscle protein catabolism through the ubiquitin–proteasome proteolytic pathway and may play a role in cancer cachexia

**DOI:** 10.1038/sj.bjc.6602725

**Published:** 2005-07-26

**Authors:** P M Sanders, S T Russell, M J Tisdale

**Affiliations:** 1 Pharmaceutical Sciences Research Institute, Aston University, Birmingham B4 7ET, UK

**Keywords:** angiotensin I/II, muscle wasting, proteasome expression, cancer cachexia

## Abstract

The ability of angiotensin I (Ang I) and II (Ang II) to induce directly protein degradation in skeletal muscle has been studied in murine myotubes. Angiotensin I stimulated protein degradation with a parabolic dose–response curve and with a maximal effect between 0.05 and 0.1 *μ*M. The effect was attenuated by coincubation with the angiotensin-converting enzyme (ACE) inhibitor imidaprilat, suggesting that angiotensin I stimulated protein degradation through conversion to Ang II. Angiotensin II also stimulated protein breakdown with a similar dose–response curve, and with a maximal effect between 1 and 2.5 *μ*M. Total protein degradation, induced by both Ang I and Ang II, was attenuated by the proteasome inhibitors lactacystin (5 *μ*M) and MG132 (10 *μ*M), suggesting that the effect was mediated through upregulation of the ubiquitin–proteasome proteolytic pathway. Both Ang I and Ang II stimulated an increased proteasome ‘chymotrypsin-like’ enzyme activity as well as an increase in protein expression of 20S proteasome *α*-subunits, the 19S subunits MSS1 and p42, at the same concentrations as those inducing protein degradation. The effect of Ang I was attenuated by imidaprilat, confirming that it arose from conversion to Ang II. These results suggest that Ang II stimulates protein degradation in myotubes through induction of the ubiquitin–proteasome pathway. Protein degradation induced by Ang II was inhibited by insulin-like growth factor and by the polyunsaturated fatty acid, eicosapentaenoic acid. These results suggest that Ang II has the potential to cause muscle atrophy through an increase in protein degradation. The highly lipophilic ACE inhibitor imidapril (Vitor™) (30 mg kg^−1^) attenuated the development of weight loss in mice bearing the MAC16 tumour, suggesting that Ang II may play a role in the development of cachexia in this model.

Cachexia is a progressive wasting syndrome, responsible for the death of about 20% of cancer patients (Inagaki *et al*, 1974), which involves substantial loss of both adipose tissue and skeletal muscle protein. Cachexia is common in many chronic or end-stage diseases such as infections, AIDS, congestive heart failure (CHF) and tuberculosis, as well as cancer. In all cases, loss of fat-free mass involves only skeletal muscle and not visceral tissues, and is reflected in an increased morbidity and mortality. In cancer cachexia, the mechanism for the selective depletion of skeletal muscle is thought to involve tumour factors, such as proteolysis-inducing factor (PIF), which inhibits protein synthesis and increases protein degradation in skeletal muscle, without affecting visceral protein reserves (Lorite *et al*, 1997). Studies in other wasting conditions suggest that other factors may be involved. Cytokines have also been shown to play an important role in muscle wasting (Zoico and Roubenoff, 2002). Thus, treatment of patients with CHF with the angiotensin-converting enzyme (ACE) inhibitor enalapril, in combination with digoxin and a diuretic, produced both increased subcutaneous (s.c.) fat and greater muscle bulk, together with a significant elevation in plasma albumin (Adigun and Ajayi, 2001). Angiotensin-converting enzyme inhibitor treatment has also been shown to halt or slow the decline in muscle strength in elderly women with hypertension and without CHF (Onder *et al*, 2002). Angiotensin-converting enzyme degrades vasodilator kinases and generates vasoconstrictor angiotensin II (Ang II). Angiotensin II has physiological effects and is capable of causing anorexia and wasting in animal models. Thus, infusion of Ang II into the rat produced a loss of between 18 and 26% of body weight by 1 week (Brink *et al*, 1996). Although 74% of this loss was attributable to a reduction in food intake, there were significant differences in body weight and lean muscle mass between Ang II-infused and pair-fed control rats. In skeletal muscle, Ang II did not significantly decrease protein synthesis, but accelerated overall protein breakdown, possibly through the ubiquitin–proteasome pathway (Brink *et al*, 2001). Further studies in rats showed that mRNA levels of the ubiquitin ligases atrogin-1 and muscle ring-finger-1 (MURF1) were upregulated in skeletal muscle by Ang II, suggesting that protein degradation was mediated through the ubiquitin–proteasome proteolytic pathway (Song *et al*, 2005). This raises the possibility that Ang II directly enhances expression and activity of this pathway, as does the tumour factor PIF (Lorite *et al*, 2001). However, the effect *in vivo* was considered to be indirect and mediated by intermediate factors such as glucocorticoids (Song *et al*, 2005). To determine whether Ang II directly accelerates protein degradation, the current study has been conducted in murine myotubes *in vitro*. To determine whether Ang II plays a role in cancer cachexia, the effect of the ACE inhibitor imidapril (Vitor™) has been evaluated on the induction of weight loss in mice induced by the MAC16 tumour.

## MATERIALS AND METHODS

### Materials

L-[2, 6-^3^H] Phenylalanine (sp. act. 48 Ci mmol^−1^), hybond A nitrocellulose membranes and enhanced chemiluminescence (ECL) development kits were from Amersham International (Bucks, UK). Foetal calf serum (FCS), horse serum (HS) and Dulbecco's modified Eagle's medium (DMEM) were purchased from Invitrogen (Paisley, Scotland). Mouse monoclonal antibodies to 20S proteasome *α*-subunits, MSS1 and p42 were from Affiniti Research Products (Exeter, UK). Mouse monoclonal antibody to myosin heavy chain was from Novocastra (Newcastle, UK). Rabbit polyclonal antisera to mouse *β*-actin and Ang I and Ang II were from Sigma Aldridge (Dorset, UK). Peroxidase-conjugated rabbit anti-mouse antibody and peroxidase-conjugated goat anti-rabbit antibody were purchased from Dako Ltd (Cambridge, UK). Both imidapril hydrochloride (Vitor™) and imidaprilat were kindly supplied by Ark Therapeutics Ltd (London, UK). Angiotensin I and Ang II receptor inhibitors were purchased from Tocris (Avonmouth, UK).

### Animals

Pure strain male NMRI mice (average weight 25 g) were obtained from our own inbred colony and transplanted with fragments of the MAC16 tumour s.c. into the flank by means of a trochar, selecting from donor animals with established weight loss, as described previously (Beck and Tisdale, 1987). Transplanted animals were fed a rat and mouse breeding diet (Special Diet Services, Witham, UK) and water *ad libitum* and weight loss was evident 10–12 days after tumour implantation without a reduction in food intake (Beck and Tisdale, 1987). When weight loss became apparent (5–7%), animals were randomised to receive imidapril hydrochloride (either 1 or 10 mg kg^−1^ dissolved in water) p.o. daily × 3 or water control. Both tumour volume and body weight were monitored daily and animals were terminated by cervical dislocation when the body weight loss reached 25%. All animal experiments followed a strict protocol, agreed with the British Home Office and the ethical guidelines that were followed meet the standards required by the UKCCR (Workman *et al*, 1998). The dimensions of the tumour were measured by callipers and tumour volume calculated from the following formula: volume=(length × width)/2.

### Cell culture

Murine C_2_C_12_ myoblasts were grown in DMEM supplemented with 10% FCS, glutamine and 1% penicillin–streptomycin under an atmosphere of 10% CO_2_ in air at 37°C. When the cells reached confluence, they were allowed to fuse to form myotubes in DMEM containing 2% HS, with medium changes every 2 days. Differentiation was complete within 5–7 days and myotubes were used experimentally no later than 4 days postdifferentiation.

### Measurement of protein degradation

#### *In vitro*

Myotubes were prelabelled for 24 h with L-[2, 6-^3^H] phenylalanine (10 *μ*Ci; sp. act. 1.32 Ci mmol^−1^) and were washed extensively prior to experimentation, as described previously (Whitehouse and Tisdale, 2003). Protein degradation was determined by the release of [2, 6-^3^H] phenylalanine into the medium after 24 h incubation with various concentrations of Ang I or Ang II in the absence or presence of inhibitors added 2 h prior to angiotensin. Cold phenylalanine (2 mM) was added to prevent reincorporation of radioactivity into the cells.

#### *Ex vivo*

Murine soleus muscles were preincubated for 45 min in 3 ml oxygenated (95% oxygen/5% carbon dioxide) Krebs–Henseleit bicarbonate buffer, pH 7.4, containing 5 mmol l^−1^ glucose and 0.5 mmol l^−1^ cycloheximide. Protein degradation was determined in the absence and presence of Ang II, with or without receptor antagonists by the release of tyrosine over a 2 h period (Waalkes and Udenfriend, 1957).

### Measurement of proteasome activity

‘Chymotrypsin-like’ enzyme activity was determined fluorimetrically by the method of Orino *et al* (1991), by the release of aminomethyl coumarin (AMC) from the fluorogenic peptide succinyl-LLVY-AMC. This method has been described previously for C_2_C_12_ myotubes (Whitehouse and Tisdale, 2003). Activity was measured in the absence and presence of the specific proteasome inhibitor lactacystin (10 *μ*M). Only lactacystin-suppressible activity was considered to be proteasome specific.

### Western blot analysis

Myotubes were incubated with various concentrations of Ang II as depicted in the figure legends, after which the medium was removed and the cells were washed with PBS and scraped from the plastic surface. They were then sonicated at 4°C in 500–2000 *μ*l of 20 mM Tris-HCl, pH 7.5, 2 mM ATP, 5 mM MgCl_2_ and 1 mM dithiothreitol. Samples of cytosolic protein (5 *μ*g), formed by centrifugation at 18 000 **g** for 5 min, were resolved on 12% sodium dodecylsulfate–polyacrylamide gels and transferred to 0.45 *μ*m nitrocellulose membranes, which had been blocked with 5% Marvel in Tris-buffered saline, pH 7.5, at 4°C overnight. The primary antibodies were used at a dilution of 1 : 1000, except for actin (1 : 200) and myosin (1 : 250), and the secondary antibodies were also used at a dilution of 1 : 2000. Incubation was for 1 h at room temperature and development was by ECL (Amersham, UK). Blots were scanned by a densitometer to quantitate differences.

### Statistical analysis

Differences in means between groups was determined by one-way ANOVA, followed by Tukey's post-test.

## RESULTS

**Figure 1:**
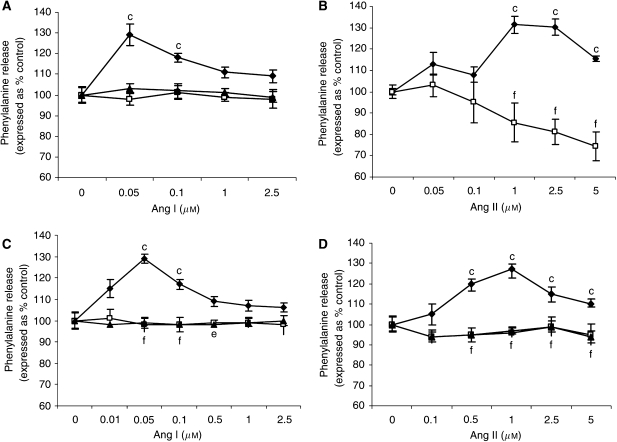
(**A**) Effect of Ang I on total protein degradation in murine myotubes in the absence (⧫) or presence of imidaprilat (50 *μ*M) (▴) or EPA (50 *μ*M) (□). (**B**) Effect of Ang II on total protein degradation in murine myotubes after 24 h incubation in the absence (⧫) or presence (□) of 50 *μ*M EPA. (**C**) Effect of Ang I and (**D**) effect of Ang II on total protein degradation in the absence (⧫) or presence of lactacystin (5 *μ*M) (▴) or MG132 (10 *μ*M) (□). Differences from control in the presence of Ang I and Ang II are indicated as c, *P*<0.001, while differences in the presence of the inhibitors are indicated as e, *P*<0.01 or f, *P*<0.001. *n*=6.

To determine a direct effect on protein degradation, Ang I was added to murine myotubes, which had been previously labelled with [^3^H] phenylalanine, and the release of radiolabel after 24 h incubation provided a monitor of total protein degradation. The results presented in Figure 1A show that Ang I significantly stimulated protein degradation, with a parabolic dose–response curve, as previously observed with PIF (Whitehouse and Tisdale, 2003), and with a maximal effect at concentrations between 0.05 and 0.1 *μ*M. The effect was attenuated by coincubation with imidaprilat (50 *μ*M), the active metabolite of the highly lipophilic ACE inhibitor imidapril (Vitor™) (Mabuchi *et al*, 1999). These results suggest that Ang I stimulates protein degradation in muscle through the conversion to Ang II. This was confirmed by the results shown in Figure 1B where Ang II also stimulated total protein degradation in myotubes, with a parabolic dose–response curve, and with a maximal effect at a concentration of 1–2.5 *μ*M. Both Ang I and Ang II increased total protein degradation by 30%, which is similar to that induced by glucocorticoids (Sacheck *et al*, 2004) and PIF (Smith *et al*, 1999). Protein degradation in myotubes initiated by both Ang I (Figure 1C) and Ang II (Figure 1D) was attenuated by the proteasome inhibitors lactacystin (5 *μ*M) and MG132 (10 *μ*M), suggesting that upregulation of the ubiquitin–proteasome proteolytic pathway was responsible for the increased protein breakdown.

**Figure 2:**
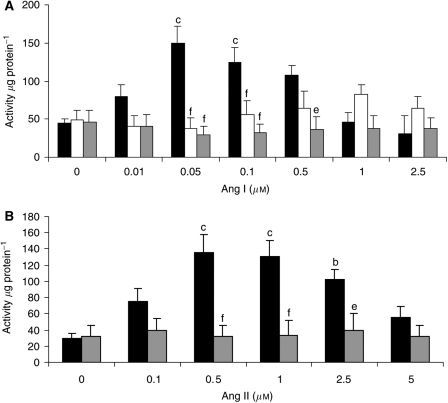
Effect of Ang I (**A**) and Ang II (**B**) on the ‘chymotrypsin-like’ enzyme activity of the proteasome in the absence (▪) or presence of imidaprilat (50 *μ*M) (□) or EPA (50 *μ*M) (▓). Differences from control in the presence of Ang I or Ang II are indicated as b, *P*<0.01 or c, *P*<0.001, while differences in the presence of inhibitors are indicated as e, *P*<0.01 or f, *P*<0.001. *n*=6.

**Figure 3:**
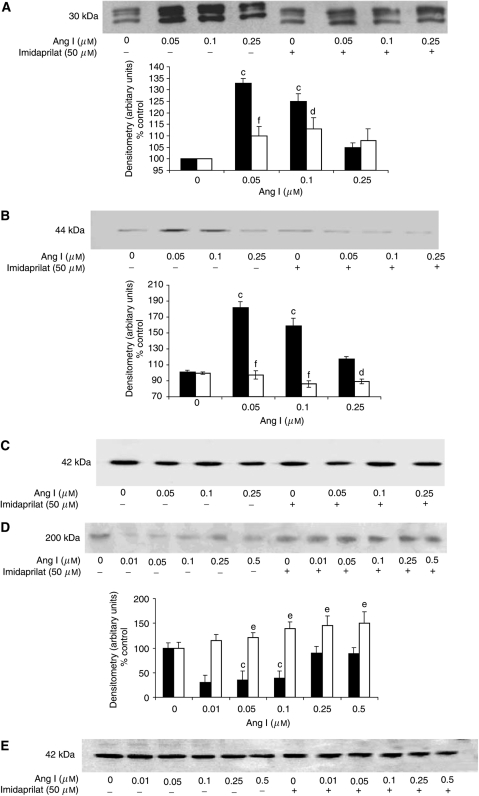
Western blots for the effect of Ang I on the expression of 20S proteasome *α*-subunits (**A**), p42 (**B**) and myosin (**D**) in soluble extracts of C_2_C_12_ myotubes after 24 h incubation alone or in the presence of imidaprilat (50 *μ*M). An actin loading control is shown in (**C**) and (**E**), and a densitometric analysis of three separate blots is shown underneath. Differences from control are shown as c, *P*<0.001, while differences in the presence of inhibitors are shown as d, *P*<0.05, e, *P*<0.01 or f, *P*<0.001.

**Figure 4:**
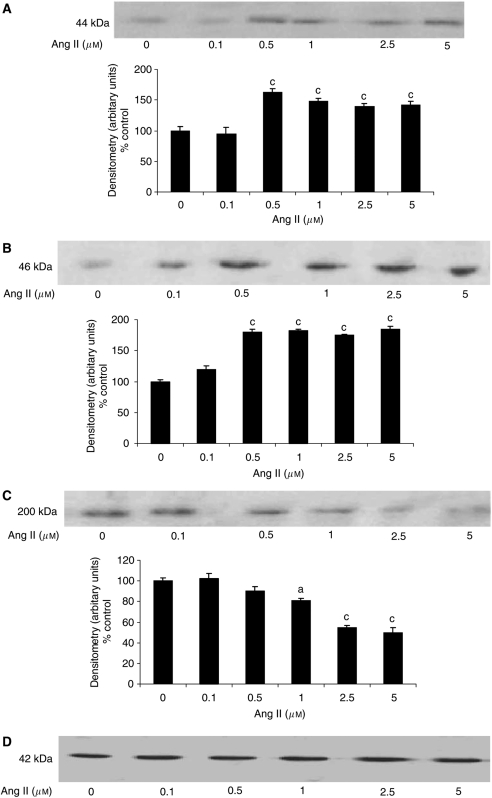
Western blots for the effect of Ang II on p42 (**A**), MSS1 (**B**) and myosin (**C**) in soluble extracts of C_2_C_12_ myotubes after 24 h incubation. An actin loading control is shown in (**D**). The blots shown are representative of three separate experiments and a densitometric analysis representing the average of the three blots is shown underneath. Differences from control are shown as a, *P*<0.05 or c, *P*<0.001.

To confirm this, the effect on proteasome functional activity was measured as the ‘chymotrypsin-like’ enzyme activity, the predominant proteasome proteolytic activity. Both Ang I (Figure 2A) and Ang II (Figure 2B) increased the proteasome ‘chymotrypsin-like’ enzyme activity with a dose–response curve similar to that for total protein degradation (Figure 1). As with total protein degradation, the effect of Ang I was attenuated by imidaprilat (Figure 2A). Western blotting showed that Ang I also induced an increase in protein expression of 20S proteasome *α*-subunits (Figure 3A), and p42, an ATPase subunit of the 19S regulator (Figure 3B), while decreasing the expression of myosin (Figure 3D). Angiotensin II also induced an increase in protein expression of p42 (Figure 4A), MSS1, another ATPase subunit of the 19S regulator (Figure 4B), and the decreased expression of myosin (Figure 4C) at the same concentrations as those inducing protein degradation, suggesting that protein breakdown was due to the induction of the ubiquitin–proteasome pathway. The effect of Ang I was attenuated by 50 *μ*M imidaprilat, confirming that it arose from the formation of Ang II (Figure 3).

**Figure 5:**
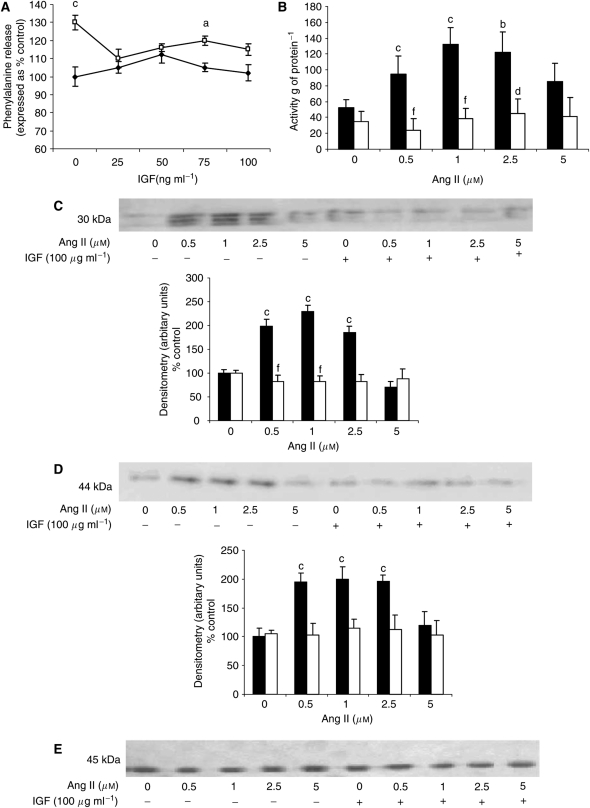
(**A**) Effect of IGF-I alone (⧫) or in combination with Ang II (1 *μ*M; □) on total protein degradation in C_2_C_12_ myotubes after 24 h incubation. (**B**) Effect of Ang II alone (▪) or in combination with IGF-I (100 ng ml^−1^) (□) on proteasome ‘chymotrypsin-like’ enzyme activity in murine myotubes. Western blots for the effect of Ang II on 20S proteasome *α*-subunits (**C**) and p42 (**D**). An actin loading control is shown in (**E**). Differences from 0 *μ*M Ang II are indicated as a, *P*<0.05, b, *P*<0.01 or c, *P*<0.001, while differences from Ang II alone are indicated as d, *P*<0.05 or f, *P*<0.001.

Since the increased protein degradation produced by Ang II has been suggested to be due to an inhibitory effect on the autocrine insulin-like growth factor (IGF-I) system (Brink *et al*, 2001), the effect of IGF-I on total protein degradation induced by Ang II (1 *μ*M) was determined in murine myotubes. Insulin-like growth factor-I completely attenuated the increase in protein degradation induced by Ang II at concentrations between 25 and 100 ng ml^−1^ (Figure 5A), and this was accompanied by complete suppression of the increase in ‘chymotrypsin-like’ enzyme activity (Figure 5B) and increase in the expression of 20S proteasome *α*-subunits (Figure 5C). These results suggest that IGF-I attenuates protein degradation induced by Ang II in skeletal muscle through downregulation of the increase in the expression of the ubiquitin–proteasome proteolytic pathway.

**Figure 6:**
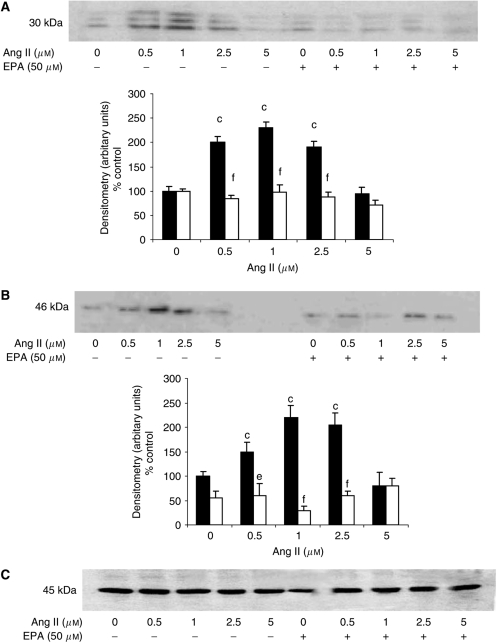
Western blot for the effect of Ang II on the expression of 20S proteasome *α*-subunits (**A**) and MSS1 (**B**) in the absence or presence of 50 *μ*M EPA. The densitometric analysis is the average of three separate blots. An actin loading control is shown in (**C**). Differences from control are indicated as c, *P*<0.001, while differences in the presence of EPA are indicated as d, *P*<0.05 or f, *P*<0.001.

In order to determine whether the mechanism of activation of protein degradation by Ang II was similar to that of PIF, protein degradation was measured in myotubes coincubated with eicosapentaenoic acid (EPA), which interrupts cellular signalling leading to an increase in proteasome expression (Smith *et al*, 1999). Stimulation of total protein degradation by both Ang I (Figure 1A) and Ang II (Figure 1B) was completely attenuated by EPA (50 *μ*M), as was also the increase in chymotrypsin-like enzyme activity (Figure 2B), and the expression of 20S proteasome *α*-subunits (Figure 6A), MSS1 (Figure 6B) and p42 (Figure 6C) as determined by Western blotting. These results suggest that the mechanism of induction of proteasome expression by Ang II is similar to that of PIF.

**Figure 7:**
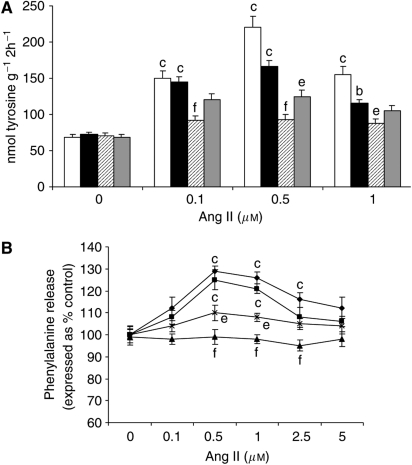
(**A**) Effect of Ang II on tyrosine release from soleus muscle, when incubated alone (□) or in the presence of 1 *μ*M of ZD7155 (▪), PD123319 (⧄) or saralasin (▓). (**B**) Effect of Ang II on total protein degradation in murine myotubes when incubated alone (⧫) or in the presence of 1 mM of ZD7155 (), PD123319 (▴) or saralasin (X). Differences from control in the presence of Ang II are shown as b, *P*<0.01 or c, *P*<0.001, while differences in the presence of inhibitors are shown as e, *P*<0.01 or f, *P*<0.001. *n*=6.

To confirm that the myotubes model was representative of skeletal muscle, the effect of Ang II on total protein degradation was determined in mouse soleus muscle *ex vivo* (Figure 7A). As in murine myotubes, Ang II directly induced protein degradation in soleus muscle, as determined by tyrosine release, and was more effective at lower concentrations than in murine myotubes. To confirm that the effect was direct and to determine the receptor involved, the protein degradation assay was performed in the presence of ZD7155, a selective competitive antagonist for the Ang II type 1 (AT_1_) receptor (Junggren *et al*, 1996) or PD123319, a selective AT_2_ receptor antagonist (Blankley *et al*, 1991), or saralasin, a competitive nonselective Ang II antagonist (Steinhausen *et al*, 1986). The results in Figure 7A show attenuation of the increased protein degradation by both PD123319 and saralasin, while ZD7155 had no effect. This suggests that Ang II induces protein degradation in skeletal muscle through interaction with the AT_2_ receptor. To confirm that the murine myotubes system was representative of skeletal muscle, the effect of the receptor antagonists on Ang II-induced protein degradation was determined in myotubes (Figure 7B). The effect was similar to that observed in soleus muscle with both PD123319 and saralasin attenuating the effect, while ZD7155 was unaffected. Thus, both in myotubes and skeletal muscle, Ang II induces protein degradation through the AT_2_ receptor, supporting the hypothesis that the effect of Ang II is a direct effect on skeletal muscle.

**Figure 8:**
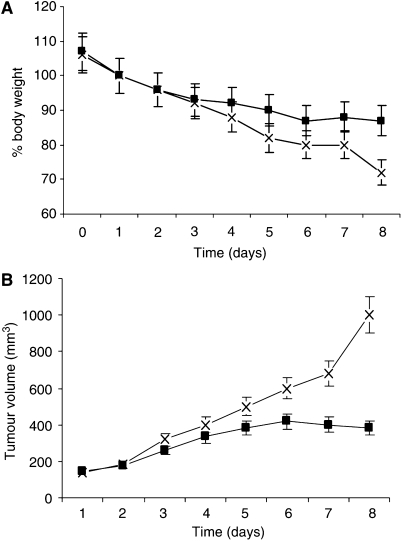
Effect of imidapril (10 mg kg × 3) (▪) on body weight (**A**) and tumour volume (**B**) in comparison with water controls (X). Imidapril was dissolved in water and was administered orally three times daily by gavage. Number of mice in each group, *n*=10 initially. In (**A**), the shapes of the curves were significantly different (*P*<0.001).

To evaluate the role of Ang II in cancer cachexia, mice bearing the cachexia-inducing MAC16 colon adenocarcinoma cachexia model were treated daily with the highly lipophilic ACE inhibitor imidapril. The reason for the choice of this particular inhibitor is that it is currently undergoing clinical evaluation for the treatment of cachexia in cancer patients. At a dose level of 30 mg kg^−1^, imidapril attenuated the development of weight loss (Figure 8A) and stabilised the increase in tumour volume (Figure 8B). The effect was dose related and was not seen with lower dose levels (3 mg kg^−1^). These results suggest that Ang II plays a role in the development of cachexia in the MAC16 model.

## DISCUSSION

This study has shown that Ang II is directly catabolic to skeletal muscle increasing intracellular protein degradation through an increased expression of the ubiquitin–proteasome proteolytic pathway. Ang I also mediates the same effects through conversion to Ang II by ACE, since the ACE inhibitor imidaprilat, the active metabolite of imidapril, in which an ethyl ester group is hydrolysed (Mabuchi *et al*, 1999), attenuates the action of Ang I. There is also evidence to suggest that formation of Ang II may contribute to the loss of body tissue in mice bearing the MAC16 tumour, since imidapril attenuated the development of weight loss in this model. Ang II directly stimulates tumour necrosis factor-*α* (TNF-*α*) and interleukin-6 (IL-6) production in human peripheral monocytes and ACE inhibitors inhibit LPS-induced TNF-*α* production (Peeters *et al*, 1998). However, the activity of imidapril in the MAC16 model could not be explained by an effect on TNF-*α* or IL-6 production, since we have previously shown no involvement of these cytokines in the cachectic process in this model (Mulligan *et al*, 1992).

Instead, Ang II directly stimulates protein degradation in myotube cultures through an increased expression and activity of key components of the ubiquitin–proteasome proteolytic pathway. The increased expression of 20S *α*-subunits would suggest a higher number of proteasomes, while the increased expression of the ATPases MSS1 and p42 provides energy for the breakdown of ubiquitinated proteins by the 26S proteasome through the hydrolysis of ATP (Coux *et al*, 1996), as well as for the assembly of the 26S proteasome through the ATP-dependent association of the 20S proteasome with the 19S regulator (Tanahashi *et al*, 1999). The decreased level of myosin in myotubes treated with Ang I/Ang II would suggest that a major component of the total protein degradation came from myofibrillar proteins. The angiotensin system initiates selective loss of myosin, while actin levels remain unchanged. A recent report (Acharyya *et al*, 2004) has shown TNF-*α* plus interferon *γ* to induce selective loss of myosin in murine myotubes, and this was also found in tibialis anterior muscle from the hind limb of cachectic mice bearing the colon 26 tumours. In both cases, actin levels remained constant. The selective loss of myosin was attributed to transcriptional repression with the cytokines, or, in the case of mice bearing colon 26 tumours, to selective degradation by the ubiquitin–proteasome pathway.

This is the first report on the ability of Ang I/Ang II to induce directly protein catabolism in isolated myotubes, although previous results (Brink *et al*, 2001; Song *et al*, 2005) from *in vivo* studies in rats suggested that Ang II induced protein degradation, but this was suggested to be due to an indirect effect. The concentration of Ang II inducing protein degradation *in vitro* is similar to that which might be achieved by direct infusion into rats (Brink *et al*, 2001). This is in the micromolar range, although normal blood levels of Ang II are below 40 pM. However, this can increase 4–5-fold in cachectic states (Coates *et al*, unpublished results) and the tissue concentrations are likely to be much higher, although they have not been measured in cachectic patients. The observation in the current study that protein degradation induced by Ang II in both soleus muscle and murine myotubes can be blocked by AT_2_ receptor antagonists, but not AT_1_, suggests that this is a physiologically relevant process.

The increase in protein degradation and ubiquitin ligases induced by Ang II *in vivo* were shown to be blocked by muscle-specific expression of IGF-I, suggesting that the downregulation of IGF-I is causally related to the muscle wasting (Song *et al*, 2005). In this study, we have shown IGF-I to attenuate completely protein degradation induced by Ang II, as well as the increase in expression and activity of the ubiquitin–proteasome proteolytic pathway, suggesting that high circulatory levels of IGF-I may be capable of suppressing the catabolic effect of Ang II. However, it is known that malnourishment associated with cancer cachexia lowers IGF-I levels (Pollak *et al*, 2004), suggesting that the catabolic effect of Ang II may be maximal in the cachectic state. The mechanism by which IGF-I attenuates the increase in proteasome expression induced by Ang II has not been evaluated, but IGF-I has been previously shown to suppress increases in C-2, -3 and -8 proteasome subunit mRNAs in the skeletal muscle of rats treated with dexamethasone as well as mRNAs for ubiquitin and E2 (Chrysis *et al*, 2002). IGF-I has been shown to suppress protein degradation in myotubes induced by dexamethasone by suppressing expression of two muscle-specific ubiquitin ligases (E3s), atrogin-1 and MURF1, which are closely associated with muscle atrophy (Sacheck *et al*, 2004). Certainly, the mechanism of induction of proteasome expression by Ang II appears to be similar to that of PIF, since the effect of both agonists is attenuated by EPA. Further studies will concentrate on the mechanism of induction of proteasome expression by Ang II.

In addition to the anticachectic effect, imidapril also produced a stabilising effect on the growth of the MAC16 tumour, which complicates the interpretation of the data on preserving body weight, since weight loss in animals bearing the MAC16 tumour is directly proportional to tumour mass (Beck and Tisdale, 1987). Other agents, for example, EPA, which attenuate cachexia in the MAC16 model, also have an inhibitory effect on tumour growth (Beck *et al*, 1991), and this has been shown to be separate from the anticachectic effect and due to starvation of the tumour of essential fatty acids such as linoleic acid (Hudson *et al*, 1993). However, Ang II is known to stimulate neovascularisation (Fernandez *et al*, 1985), which in some cases is a requirement for tumour growth, while *in vitro* it stimulates cell replication in the absence of blood vessels (Paquet *et al*, 1990). ACE inhibitors retard growth of cancer cells *in vitro* (Reddy *et al*, 1995) and inhibit tumour growth *in vivo* (Hii *et al*, 1998), possibly through an effect on angiogenesis, while long-term use of ACE inhibitors may protect against the development of cancer (Lever *et al*, 1998). These results suggest that imidapril may inhibit growth of the MAC16 tumour by a mechanism unrelated to its effect on cachexia, and thus it is not possible to determine unequivocally whether imidapril has a direct anticachectic effect in this model.
